# 局限期小细胞肺癌的治疗进展

**DOI:** 10.3779/j.issn.1009-3419.2011.10.08

**Published:** 2011-10-20

**Authors:** 敬慧 王, 树才 张

**Affiliations:** 101149 北京，首都医科大学附属北京胸科医院肿瘤内科 Department of Medical Oncology, Beijing Chest Hospital, Capital Medical University, Beijing 101149, China

小细胞肺癌（small cell lung cancer, SCLC）占全部肺癌的15%-20%。其分期系统有两种，临床上最常用的是美国退伍军人医院分期系统，将SCLC分为局限期和广泛期：病变局限于同一侧胸腔并可安全地包括在一个可耐受的放射野内为局限期（limited-disease small cell lung cancer, LD-SCLC）；病变超出同一侧胸腔，包括恶性胸腔、心包积液及远处转移为广泛期（extensive-disease small cell lung cancer, ED-SCLC）。第二种是第7版TNM分期。临床上制定治疗决策更多的是依据退伍军人医院分期法。此外，在SCLC的临床试验中，对侧纵隔和同侧锁骨上淋巴结转移被视为局限期，对侧肺门和对侧锁骨上淋巴结转移被视为广泛期。

SCLC倍增时间短，更易早期发生远处转移，确诊时仅1/3患者为局限期。尽管对初始化疗和放疗高度敏感，但大多数LD-SCLC患者远期生存差，5年生存率约为12%-17%。近些年来，随着新的药物出现和综合治疗模式的不断探索，小细胞肺癌的治疗有了一些新的变化，本文将就当前LD-SCLC的治疗及其进展作一综述。

## 1 化疗

### 1.1 一线化疗

2002年发表的一项Ⅲ期试验结果^[1]^显示，局限期患者接受顺铂+依托泊苷（EP）方案治疗后的中位生存期（median survival time, MST）、2年生存率、5年生存率明显优于环磷酰胺/阿霉素/长春新碱（CAV），分别为14.5个月、9.7个月，25%、8%，10%、3%，EP疗效优于CAV，化疗副反应也低。因此，EP取代CAV成为局限期患者治疗的首选，也是NCCN指南推荐用于LD-SCLC的一线方案。

近几年，许多临床试验探索了一些新药在ED-SCLC患者中的一线治疗价值，如拓扑替康、伊立替康、培美曲塞等，但针对LD-SCLC患者的一线化疗方案的临床试验很少。迄今，EP仍是LD-SCLC的标准方案，尚未被其它方案取代。

临床上，为了减轻顺铂的消化道、神经及肾脏毒性，常用卡铂替代顺铂，但卡铂的血液学毒性高于顺铂，卡铂能否取代顺铂治疗局限期患者还未得到充分地评价。因此，仅应对有顺铂用药禁忌或耐受性差的局限期患者使用卡铂。与放疗同步时，推荐使用EP方案。

老年患者的比例处于上升趋势，监测、流行病学和最终结果分析（Surveillance, Epidemiology and End Results, SEER）显示新确诊的SCLC中>70岁的患者占32%，>80岁的患者约占10%。当前针对老年患者的随机对照试验并不多，多数为回顾性研究。在Siu等^[2]^报告的一项回顾性研究中，对纳入的520例 < 70岁局限期患者和88例≥70岁患者采用CAV/EP化疗联合放疗，两组的有效率（response rate, RR）分别为78%、82%（*P*=0.05），5年生存率分别为11%、8%（*P*=0.14），老年患者与较年轻患者的预后相似。Jara^[3]^与Noguchi^[4]^的研究与Siu的结果一致。Caprario等^[5]^将SEER数据库中10, 428例≥65岁SCLC患者作了回顾性分析，67.1%的患者接受化疗，39.1%放疗，3.4%手术，21.8%未治疗，使用最多的方案是依托泊苷联合顺铂或卡铂。与65岁-69岁患者比较，>85岁的患者明显不愿接受化疗，MST为7个月。预后好的因素有女性、黑人、病变局限、接受任何治疗、合并症分低。此项分析证实老年患者能从治疗中获益。

老年患者作为一个特殊人群，具有独特的生理和器官功能特点。临床制定治疗策略时，除了要考虑年龄，更要重视患者的实际体力功能状态。如果患者具备日常生活能力，可以接受联合化疗（如果适合，也可行放疗），但需注意的是，骨髓抑制、乏力、器官储备低在老年患者中更常见。

### 1.2 二线化疗

大多数SCLC患者在一线治疗后都会复发或进展，临床上将复发患者分为以下3类：一线治疗过程中进展为难治性复发；一线治疗结束后3个月内进展为耐药复发；一线治疗结束后3个月以上进展为敏感复发。不同复发患者对二线化疗药物的疗效差异较大。难治或耐药复发患者对大多数药物或方案的疗效差（≤10%），敏感复发的预期有效率约为25%，复发时间距一线治疗结束6个月以上患者的有效率约为57%。复发患者的预后差。

一项Ⅱ期试验证实，伊立替康、紫杉醇、拓扑替康、氨柔比星等是有效的二线治疗药物（表 1）。Belotecan和吡铂也显示出一定的疗效，有关Ⅲ期试验正在进行中。两项Ⅱ期试验证实培美曲塞单药二线治疗SCLC无效，一项Ⅱ期研究^[6]^显示吉西他滨单药的疗效也很低。

**1:** 单药或联合方案治疗复发小细胞肺癌的前瞻性研究 Prospective studies of single-agent or combination chemotherapy for relapsed small cell lung cancer

Regimen	*n*	Refractoy patients (%)	RR (%)	RR in refractory patients (%)	MST (month)
Irinotecan	15	7	47	NA	6.2
Irinotecan	31	64	0	0	4.6
Paclitaxel	24	NA	29	NA	3.3
Picoplatin	77	92	4	NA	5.2
Belotecan	25	NA	24	NA	9.9
Cisplatin+Irinotecan	24	64	80	81	7.9
Etoposide+Irinotecan	25	16	71	75	9.0
Carboplatin+Paclitaxel	34	NA	74	NA	7.2
Carboplatin+Paclitaxel	32	100	25	25	7.0
RR: response rate; MST: median survival time; NA: not available.					

拓扑替康为拓扑异构酶Ⅰ抑制剂，一项Ⅱ期研究显示拓扑替康单药治疗敏感复发SCLC的RR为14.0%-37.8%，MST为6.0个-8.7个月，耐药复发患者的RR为2.4%-11.0%，MST为3.8个-4.7个月。基于Von Pawel等^[10]^的一项Ⅲ期随机试验的结果，拓扑替康成为唯一一个被美国FDA批准用于对一线化疗敏感、2个-3个月以后进展的SCLC的二线治疗药物（表 2）。

**2:** 拓扑替康治疗复发SCLC的随机临床试验 Randomized studies of single-agent topotecan for relapsed SCLC

Study	Phase	Regimen	*n*	RR (%)	MST (week)
Von Pawel^[7]^	Ⅱ	Oral topotecan	52	23.0	32.0
		iv topotecan	54	15.0	25.0
Eckardt^[8]^	Ⅲ	Oral topotecan	153	18.3	33.0
		iv topotecan	151	21.9	35.0
O’Brien^[9]^	Ⅲ	BSC	67	–	13.9
		Topotecan	70	7.0	25.9
Von Pawel^[10]^	Ⅲ	CAV	104	18.3	24.7
		Topotecan	107	24.3	25.0
iv: intravenous injection; BSC: best support care; CAV: CTX/ADM/VCR.					

氨柔比星是一种新的蒽环类抗生素，通过抑制拓扑异构酶Ⅱ的活性抗肿瘤细胞增殖。Ⅱ期试验证实其无论对敏感复发或耐药复发SCLC患者均有很好的疗效（表 3）。

**3:** 氨柔比星单药或联合方案治疗复发SCLC的研究 Single-agent amrubicin or combination chemotherapy for relapsed SCLC

Study	Phase	Dose (mg/m^2^)	Chemosensitivity	*n*	RR (%)	MST (month)
Kato^[11]^	Ⅱ	45, d1-d3	S	24	50.0	10.4
			R	10	60.0	6.8
Onoda^[12]^	Ⅱ	40, d1-d3	S	44	52.0	11.6
			R	16	52.0	10.3
Kaira^[13]^	Ⅱ	35, d1-d3	S	10	60.0	12.0
			R	19	36.8	11.0
Ettinger^[14]^	Ⅱ	40, d1-d3	S	0	-	-
			R	75	21.3	6.0
Hirose^[15]^	Ⅱ	30, d1-d3, carboplatin AUC=4	S	12	58.4	10.0
			R	15	15.4	5.0
S: sensitive; R: resistant; AUC: area under the curve.						

除了上述Ⅱ期临床试验结果以外，Jotte等^[16]^在2011年ASCO会议上报告了一项比较氨柔比星与拓扑替康二线治疗SCLC的Ⅲ期随机临床试验结果，637例患者随机分为氨柔比星组（424例，40 mg/m^2^，d1-d3）或拓扑替康组（213例，1.5 mg/m^2^，d1-d5），试验的后1/3阶段患者接受预防性G-CSF治疗。氨柔比星组和拓扑替康组的总有效率（overall response rate, ORR）、无进展生存期（progression-free survival, PFS）、总生存期（overall survival, OS）分别为31%、17%（*P*=0.000, 2）；4.1个月、4.0个月（*P*=0.98）；7.5个月、7.8个月（*P*=0.17）。两组12个月生存率无差异，分别为28%、25%；氨柔比星组的18个月及24个月的生存率明显高于拓扑替康组，分别为16%、9%（*P*=0.045）；9%、3%（*P*=0.049）。亚组分析，两组在敏感复发患者中的OS无差异，氨柔比星在耐药复发患者的OS明显优于拓扑替康组（6.2个月、5.7个月，*P*=0.047），12个月和18个月的生存率也明显优于拓扑替康组。氨柔比星组3/4级血液学毒性明显低于拓扑替康组。这个Ⅲ期试验证实氨柔比星在二线治疗方面优于拓扑替康，尤其是对耐药复发患者。多项试验证实氨柔比星是当前SCLC二线治疗中最有前景的细胞毒药物。

氨柔比星最常见的毒性是骨髓抑制，单药3级-4级骨髓抑制发生率高。Onoda等^[12]^的研究发现粒细胞减少发生率为83%，血小板减少发生率为20%，贫血发生率为33%。Ettinger等^[14]^的研究发现粒细胞减少发生率为67%，血小板减少发生率为41%，贫血发生率为30%。

## 2 放疗

### 2.1 胸部放疗

包括2, 000多例患者的两项*meta*分析^[17, 18]^显示，放疗能使局限期患者的局部复发率降低25%-30%，2年生存率提高5%-7%，联合放疗能延长局限期患者的生存期，放疗在局限期患者的治疗中起着非常重要的作用。

放疗实施过程中需考虑化疗和放疗的顺序（同步、序贯、交替）、放疗时间（早期、后期）、放疗剂量、分割方式及放疗靶区（治疗前、治疗后缩小的肿瘤）等因素。已有较多大型临床研究针对这些不同的治疗选择作了探索。日本肿瘤协作组实施了一项比较同步和序贯放疗的临床试验^[19]^，共入组231例患者，均接受EP化疗4周期，放疗剂量为45 Gy、1.5 Gy/次、2次/日，同步组放疗于第1周期第2天开始，序贯组放疗于第4周期第85天开始。同步组与序贯组的OS、2年生存率、3年生存率分别为27.2个月、19.7个月（*P*=0.097）；54.4%、35.1%；29.8%、20.2%。两组生存虽无统计学差异，但同步放疗组结果优于序贯组，试验支持同步放疗。另外，一项EORTC的研究^[20]^比较了交替与序贯放疗对LD-SCLC的疗效，结果显示两组3年生存率无差异，分别为12%、15%，但交替放疗组的血液学毒性更高。Lebeau等^[21]^也比较同步与交替放疗治疗局限期患者的疗效，两组也无差异，MST分别为13.5个月和14个月。

加拿大国家癌症研究所开展的一项研究^[22]^比较了早期与后期放疗的差异，早期放疗于第2周期开始，后期放疗于第6周期开始，结果证实早期同步放疗在提高局部控制、全身控制及延长生存期方面均优于后期同步放疗，MST、2年生存率、3年生存率分别为21.2个月、16个月；40%、33.7%；29.7%、21.5%（*P*=0.008）。由Jeremic等^[23]^报告的结果也支持早期放疗。还有两项针对放疗时间的meta分析，第一项是Fried的研究^[24]^，纳入了7项随机研究的1, 524例患者，对后期放疗（化疗开始9周或第3周期结束）及早期放疗（化疗开始9周以内）进行比较，早期同步放疗给患者带来小而明显的生存期延长，2年生存率提高5%（*P*=0.03），3年生存率也显示出早期同步有优势，亚组分析中，超分割早期同步组的2年、3年生存率均明显优于后期同步。第二项*meta*分析由Ruysscher等^[25]^报告，早期放疗（化疗开始后30天内）与后期放疗的2年生存率、5年生存率无差异，但如果去除其中仅有的一个非铂化疗方案的试验，早期放疗组的5年生存率明显优于后期放疗组（分别为20.2%、13.8%）。

东方肿瘤协作组/放射肿瘤治疗组开展了一项大样本研究^[26]^，比较EP联合1次/日或2次/日放疗的疗效。417例局限期患者接受同步化放疗，总量45 Gy、1次/日，共5周，或2次/日，共3周。2次/日组和1次/日组的MST分别为23个月、19个月（*P*=0.04），5年生存率分别为26%、16%，2次/日组患者的生存期更长，但3级-4级食管炎比例也更高（27% *vs* 11%, *P* < 0.001）。两组的局部复发率无差异（1次/日组为52%，2次/日组为36%，*P*=0.066）。这项Ⅲ期试验是证实2次/日放疗能延长生存期的里程碑，但这项试验中1次/日的放疗剂量未给到到最大耐受剂量，因此，在生物学剂量相等的情况下，超分割是否优于1次/日放疗仍未明确。实际上，总量45 Gy、2次/日的放疗模式在临床实践中并未得到很多的应用，2003年Movsas^[27]^研究显示，能够接受这种放疗模式的局限期患者不足10%，>80%的患者还是接受1次/日放疗模式。鉴于2次/日放疗的副反应更重，选择接受2次/日放疗同步化疗的患者应具备良好的PS评分和基础肺功能。

为了探索LD-SCLC最佳的放射剂量和分割方式，2008年美国、欧洲和加拿大分别启动了两项Ⅲ期临床试验，一项为CALGB 30610试验，随机分为3组，A组为45 Gy（3周）、2次/日，B组为70 Gy（7周）、1次/日，C组为61.2 Gy、缩野加量（5周），B组和C组中出现急性副反应大的一组将停止，试验继续2组比较，放疗于化疗第1周期开始，化疗方案为EP。另一项为CONVERT试验，随机分为2组，A组为45 Gy、2次/日（3周），B组为66 Gy、1次/日（6.5周），放疗于化疗第2周期开始，化疗方案也为EP^[28]^，这两项试验仍在进行中。

有临床研究^[29-31]^结果显示，以治疗前病变为靶区和以治疗后病变为靶区的局部复发率无差异，均约为30%。这3项试验的不足是研究时间较早，当时还未普及采用CT制定放疗计划的技术。

### 2.2 预防性脑放疗（prophylactic cranial irradiation, PCI）

50%的LD-SCLC在一线治疗缓解后发生脑转移，明显影响患者的生活质量和预后。20世纪80年代初，有随机试验证实PCI可有效降低脑转移的发生率，但多数单个研究未证明患者能获得明显生存优势。在此背景下，Auperin等^[32]^进行了一项*meta*分析，共纳入7项研究，987例完全缓解的SCLC患者分为PCI组（526例）或对照组（461例），其中局限期患者占85%，分析发现PCI使3年脑转移的发生率降低25.3%（对照组为58.6%，PCI组为33.3%，*P* < 0.001），PCI使患者3年生存率提高5.4%（对照组为15.3%，PCI组为20.7%，*P*=0.01），证实PCI能预防脑转移，延长患者生存期。这项*meta*分析使PCI成为完全缓解或缓解好的SCLC的标准治疗。近来的一项回顾性研究^[33]^共入组7, 995例LD-SCLC，其中670例患者在一线治疗后接受PCI，接受PCI的局限期患者2年生存率、5年生存率、10年生存率优于未接受PCI患者，分别为42%、19%、9%和23%、11%、6%（*P* < 0.001），也证实PCI可延长患者生存期。

为探索PCI的最适剂量，PCI合作组开展了一项国际研究^[34]^，共入组来自22个国家157个中心的720例局限期患者，将一线化放疗后获得完全缓解的患者随机分为高剂量组（360例，36 Gy）或标准治疗组（360例，25 Gy/10f），高剂量组和标准剂量组的2年脑转移发生率分别为23%、29%（*P*=0.08），2年生存率分别为37%、42%（*P*=0.05），两组脑转移发生率和生存率无差异，高剂量组并无优势，但高剂量组的副反应更大，乏力、头痛、恶心呕吐发生率分别为34%、28%、28%，而标准剂量组为30%、24%、23%。因此，25 Gy/10f是局限期化疗后获得缓解患者PCI的标准剂量。

PCI的急性副反应包括乏力、头痛、恶心呕吐，这些都是可控的。远期副反应表现为神经毒性，在放疗单次剂量≥3 Gy或与化疗同步进行时明显。一项小样本发现患者远期出现记忆力下降、智力受损、痴呆、共济失调等副反应。

Le Péchoux等^[35]^经过3年的观察发现，高剂量组与标准剂量组患者在17项评价生活质量的指标上均无明显差异，两组均表现出交流缺陷、下肢乏力、智力和记忆力下降轻微加重。临床上医生应告知患者PCI的副反应及其延长生存和降低脑转移的益处。PCI不推荐用于PS差（3分-4分）或有精神功能受损的患者。

## 3 手术

由于放化疗是SCLC患者非常有效的治疗手段，因此手术在SCLC中的作用一直是有争议的。仅有的一项随机研究^[36]^结果显示患者未从手术中获益，但这项研究较早，而且试验设计也存在一些不足，很可能影响了试验结果。虽然局限期患者从放化疗能获得很好的疗效，仍不能解决局部病灶的高复发率，所以手术切除是一个能够更好控制局部复发的手段。有几项非随机临床研究显示手术作为联合治疗的一部分，能够提高局部控制率，改善预后。

Fujimori等^[37]^报告一组Ⅰ期-Ⅱ期和Ⅲ期患者接受诱导化疗和手术后，3年生存率分别为73.3%、42.9%（*P*=0.018）。Brock等^[38]^报告Ⅰ期SCLC患者手术后辅助化疗，5年生存率为58%。Tsuchiya等^[39]^报告Ⅰa期患者完全切除后行辅助化疗，并接受PCI，5年生存率为73%。PCI能降低脑转移发生率，进一步延长生存期，推荐辅助化疗后行PCI治疗。Koletsis等^[40]^对PubMed和Embase两个数据库中1980年-2009年间有关SCLC手术的研究进行回顾性分析，认为对于T1-2N0 SCLC患者适合手术联合术后化疗。

国际肺癌研究协会分期数据库中有12, 620例SCLC，其中349例SCLC患者进行了手术切除。按照第7版TNM分期，完全切除的SCLC的5年生存率分别为：Ⅰa期56%、Ⅰb期57%、Ⅱa期38%、Ⅱb期40%、Ⅲa期12%、Ⅲb期为0，手术患者的生存率更高^[41]^。另外Yu等^[42]^分析了1998年-2004年间国家癌症研究所SEER数据库中247例接受手术切除患者的结果，其中205例Ⅰ期患者只接受肺叶切除，5年生存率为50.3%，优于同为Ⅰ期未手术的患者，与国际肺癌研究协会中的结果一致。

Schreiber等^[43]^将1988年-2002年SEER登记的LD-SCLC患者（T1-T2Nx-N0）或病变局限患者（T3-T4Nx-N0）作了分析，共14, 179例，其中863例进行手术切除，手术在T1/T2患者中最多。T1-T2Nx-N0患者手术后的MST从15个月提高到42个月（*P* < 0.001），T3-T4Nx-N0患者MST从12个月提高到22个月（*P* < 0.001）。肺叶切除患者的预后最好（*P* < 0.001）。局限期患者经肺叶切除的MST为65个月，5年生存率为52.6%，病变局限患者MST为25个月，5年生存率为31.8%。分析证实，手术，尤其是肺叶切除，可明显延长经选择的局限期患者生存期。

Tashi等^[44]^在2011年ASCO上报告了一项回顾性研究，来自VA中心癌症登记库的8, 791例LD-SCLC患者中，915例患者接受外科切除，Ⅰ期占65.8%，Ⅱ期占13.4%，Ⅲ期占20.8%。424例接受肺叶切除及淋巴结清扫术，193例行局部切除，188例行简单肺叶切除，64例行肺切除。结果显示，手术组的各期患者的生存均高于未手术患者，Ⅰ期MST为45.9个月*vs* 15.9个月，Ⅱ期MST为39.4个月*vs* 13.7个月，Ⅲ期MST为21.8个月*vs* 11.5个月（*P* < 0.000, 1）。可见，手术能提高各分期患者的生存期。

上述几项大样本的回顾性研究显示早期（T1-2N0）或病变局限的患者、甚至是Ⅲ期患者是可能从包括手术在内的综合治疗中获益的。Ⅰ期患者手术后推荐化疗，同时根据淋巴结情况决定是否放疗，辅助治疗结束后推荐患者行PCI。对于Ⅰ期患者是首选手术还是先诱导化疗后手术，还是有争议的。如果对于Ⅱ期、Ⅲ期患者实施手术，必须以多学科综合治疗为基础，在临床对照试验背景下进行。然而，这些均为回顾性研究，证据级别相对较低。因此，当前急需开展前瞻性多中心随机对照研究明确手术在LD-SCLC中的作用。

## 4 靶向治疗

近些年来，靶向药物越来越多地应用于恶性肿瘤的治疗，现也有许多临床试验探索靶向治疗在SCLC患者中的作用，多数试验是在广泛期患者进行，局限期患者中的靶向治疗试验较少。

### 4.1 抗血管药物

#### 4.1.1 贝伐珠单抗

贝伐珠单抗是抑制血管内皮生长因子的人源化单克隆抗体，已用于非小细胞肺癌和乳腺癌等肿瘤的治疗。一项Ⅱ期试验^[45]^评价了LD-SCLC放化疗后采用贝伐珠单抗维持治疗的疗效，共入组57例LD-SCLC，采用卡铂联合伊立替康化疗4周期，放疗61.2 Gy，疾病未进展患者接受贝伐珠单抗治疗（10 mg/kg，1次/2周，共10次）。结果显示，缓解率为80%，1年、2年生存率分别为71%、29%，与EP联合放疗效果相似。该作者开展的另一项贝伐珠单抗联合化疗治疗局限期患者的试验正在进行中。另一项试验^[46]^采用紫杉醇联合贝伐珠单抗二线治疗敏感复发SCLC，共入组34例，紫杉醇90 mg/m^2^，d1，8，15，贝伐珠单抗10 mg/kg，d1，d 15，4周重复。结果显示，中位PFS为14.7周，ORR为18.1%，疾病稳定（stable disease, SD）占39.3%，紫杉醇联合贝伐珠单抗并未改善敏感复发SCLC预后。

#### 4.1.2 范德他尼

范德他尼是作用于VEGFR-2、VEGFR-3、RET、EGFR/HER1的口服抗血管药物。在一项Ⅱ期试验^[47]^中，与安慰剂作对照，107例局限期和广泛期患者接受范德他尼300 mg、qd进行维持治疗，试验组与对照组的PFS和OS分别为2.7个月、2.6个月；11.9个月、10.6个月，两组无差异。与对照组比较，局限期患者生存明显延长，广泛期患者生存期缩短。

#### 4.1.3 索拉非尼

索拉非尼是作用于肿瘤增殖及抗血管通路的口服多激酶抑制剂。SWOG开展了一项研究评价其在二线治疗中的作用。一线含铂化疗进展的81例患者口服索拉非尼400 mg、2次/日、28天，18例（22%）因副反应未继续治疗。3例部分缓解（partial response, PR）（4%），25例SD（32%）。铂类敏感患者的MST为7个月，铂类耐药的MST为5个月，与当前的细胞毒药物疗效相似。主要的3级-4级毒性包括皮疹、流感样症状、代谢异常。因索拉非尼毒性发生率高，超过20%的患者停止治疗，同时并未显示出更好的疗效，但SWOG研究者仍推荐在SCLC中进一步评价索拉非尼的作用^[48]^。

#### 4.1.4 沙利度胺

沙利度胺是一种谷氨酸衍生物，具有抗炎及可能的抗血管作用。有两项大的Ⅲ期试验评价了沙利度胺联合化疗治疗SCLC的作用，第一项试验^[49]^是在724例LD-SCLC和ED-SCLC中比较卡铂+依托泊苷联合或不联合沙利度胺的疗效，试验组和对照组的OS分别为10.1个月和10.5个月，两组无差异。沙利度胺增加血栓事件发生（沙利度胺组为19%、安慰剂组为10%）。第二项Ⅲ期试验^[50]^是在广泛期患者中进行，OS仍无差异。试验证实沙利度胺未显示对SCLC的优势。

#### 4.1.5 舒尼替尼

舒尼替尼是多靶点的酪氨酸激酶抑制剂。2011年ASCO会议上Han等^[51]^报告了舒尼替尼治疗复发或难治SCLC的Ⅱ期试验，25例患者入组，23例可评价。ORR为9%（2例敏感复发患者达PR），SD为30.4%。PFS和OS分别为1.4个月、5.6个月。3级-4级毒性包括血小板减少（63%）、粒细胞减少（25%）、乏力（8%）、食欲下降（8%）。2级甲状腺功能低下和高血压发生率分别为42%、17%，46%患者需要1次-2次减量。试验未达到主要终点，且耐受性差，未能为进一步研究提供依据。

### 4.2 基质金属蛋白酶（matrix metalloproteinases, MMP）抑制剂

Marimistat是第一个进入临床试验的口服MMP抑制剂，在一项随机双盲安慰剂对照的Ⅲ期试验^[52]^中，共532例LD-SCLC或ED-SCLC患者一线治疗后接受Marimistat维持治疗（10 mg，2次/日，服用2年），试验组和安慰剂组的OS分别为9.7个月和9.3个月，试验组的生活质量差。其它MMP抑制剂试验的结果也令人失望。

### 4.3 其它靶向药物

如mTOR抑制剂、kit抑制剂、Bcl-2抑制剂及EGFR-TKI等在广泛期患者中进行了多次临床试验，患者均无获益。

## 5 小结

EP联合放疗获得完全或部分缓解后接受PCI治疗是LD-SCLC的标准治疗。推荐放疗与化疗早期同步进行，但最佳的分割模式和剂量还有待开展大样本的随机试验来明确。二线治疗总体疗效较低，患者的耐受性也明显下降，靶向治疗仍是未来值得探索的研究方向。同时，应开展前瞻性随机对照试验评价手术在LD-SCLC综合治疗中的地位。
